# The Influence of the Coronavirus Disease 2019 Pandemic on Influenza Vaccination Refusal and Patient Satisfaction

**DOI:** 10.1093/ofid/ofaf351

**Published:** 2025-06-18

**Authors:** Olivia M Man, Jack W McHugh, Jeremy Young, Laurie L Wilshusen, Lacey Hart, Tripp Welch, John C O’Horo, Douglas W Challener

**Affiliations:** Department of Internal Medicine, Mayo Clinic School of Medicine and Science, Rochester, Minnesota, USA; Division of Public Health, Infectious Diseases, and Occupational Medicine, Mayo Clinic, Rochester, Minnesota, USA; RavenRock Innovations, Highlands Ranch, Colorado, USA; Mayo Clinic Quality, Rochester, Minnesota, USA; Mayo Clinic Quality, Rochester, Minnesota, USA; Mayo Clinic Quality, Rochester, Minnesota, USA; Division of Public Health, Infectious Diseases, and Occupational Medicine, Mayo Clinic, Rochester, Minnesota, USA; Division of Public Health, Infectious Diseases, and Occupational Medicine, Mayo Clinic, Rochester, Minnesota, USA

**Keywords:** COVID-19, Engagement, Vaccination

## Abstract

**Background:**

We examined how the COVID-19 pandemic influenced influenza vaccination, healthcare engagement, and patient satisfaction.

**Methods:**

We retrospectively analyzed influenza vaccination records and patient experience surveys of adult primary care patients in southeastern Minnesota during two 3-year phases: Prepandemic (1 January 2017–28 February 2020) and Pandemic-Plus (1 March 2020–31 December 2023). Vaccination status was defined as “always vaccinated” (AV), “never vaccinated” (NV), or “intermittently vaccinated” (IV) for seasonal influenza.

**Results:**

During the Pandemic-Plus phase, 7.0% (n = 3556) of the Prepandemic AV and 48.7% (n = 16 710) of the Prepandemic IV patients never received another influenza vaccine. Compared to AV, IV and NV patients were more likely to reside in areas with greater socioeconomic deprivation (odds ratio [OR], 1.58 [95% confidence interval {CI}, 1.53–1.62] and 1.99 [1.94–2.05], respectively), have a high school education or less (2.86 [2.74–2.98] and 3.38 [3.23–3.53]), and report healthcare disengagement (1.59 [1.55–1.64] and 4.21 [4.09–4.33]). After adjusting for Area Deprivation Index and medical comorbidities, healthcare disengagement increased among the NV versus AV between phases (3.33 [3.24–3.41] and 4.23 [4.10–4.35]). In a subgroup analysis those with severe comorbidities were less likely AV (NV vs AV: 1.21 [1.14–1.27]) and more dissatisfied with care (NV vs AV: 1.25 [1.18–1.33]).

**Conclusions:**

The COVID-19 pandemic altered vaccination behaviors and healthcare satisfaction, especially among those at high risk of developing influenza-related complications. Medical providers and public health officials should be aware of factors associated with vaccine refusal to better target interventions.

## Teaser

We observed a decline in vaccination rates and patient satisfaction following the COVID-19 pandemic, particularly among those with severe health comorbidities. Decline in vaccination is associated with residing in areas with high deprivation, self-identifying as Black, and having a lower education level.

The coronavirus disease 2019 (COVID-19) pandemic exposed significant challenges in patient adherence to medical guidelines, including completion of routine vaccinations. Seasonal influenza vaccination coverage in the United States has declined since 2020, according to estimates from the Centers for Disease Control and Prevention [1]. Concerningly, this decline is thought to be highest among those at the greatest risk of developing serious influenza-related complications [1]. The burden of seasonal influenza varies by year but continues to place a significant strain on the health system—contributing to an estimated 710 000 hospitalizations and 51 000 deaths each year [2]. Vaccination can mitigate this burden, offering direct individual and indirect community protection through reducing transmission rates [3, 4].

Challenges with declining vaccination rates are further complicated by vaccine hesitancy, a complex phenomenon defined by the World Health Organization as “a delay in acceptance or refusal of vaccination despite availability of vaccine services. It is influenced by factors such as complacency, convenience and confidence” [5, 6]. While research related to vaccine hesitancy has traditionally focused on childhood vaccines [7], increasing attention is being directed toward its spillover effects among adults [6, 8], which has been associated with misinformation [9, 10], safety concerns [9, 11], and one's social network [12]. Moreover, although not all patients who exhibit vaccine hesitancy refuse vaccinations, a meta-analysis found an association between vaccine hesitancy and vaccination refusal [13].

The emergence of severe acute respiratory syndrome coronavirus 2 (SARS-CoV-2) and subsequent availability of vaccines has highlighted the need to better understand adult vaccination behaviors. Vaccine uptake has previously been associated with sociodemographic factors, including race [6, 14–16], age [16, 17], location [7], political beliefs [6, 16, 18], education level [17, 19, 20], health status [17], and income [17, 21], but a meta-analysis has found that these are inconsistent predictors [22]. Understanding how the COVID-19 pandemic has influenced these patterns is crucial to informing future vaccination strategies.

While vaccine hesitancy and refusal are commonly attributed to low confidence in the health system and negative vaccine perceptions [22, 23], previous research has focused on health system–led vaccination interventions, including personalized communications from physicians to patients, provider-led education programs, and increasing accessibility [24, 25]. The role of patient satisfaction in shaping confidence in the health system—and, consequently, the willingness to engage with vaccination interventions—remains poorly understood.

This study examines how the COVID-19 pandemic influenced seasonal influenza vaccination refusal among adults in our health system. We identify how influenza vaccination rates changed over space and time and assess the relationship between influenza vaccination status, sociodemographic factors, and patient satisfaction. Finally, an interrupted time series analysis evaluates how key pandemic events influenced influenza vaccination rates.

## METHODS

### Study Site and Population

We retrospectively analyzed influenza vaccination records of adult primary care patients within our Minnesota health system (Institutional Review Board number 23-003974). All patients aged ≥18 years who had seen a primary care physician at any time between 2017 and 2023 were included. Patients were not excluded based on frequency of visits as it is a marker of healthcare engagement. Patients must have resided in the catchment area to establish primary care. The seasonal influenza vaccine is recommended for all adult patients [3], is available at all of the primary care locations included in the study, and is covered by health insurance plans. Our health system, located in southeastern Minnesota, contains both moderately sized cities as well as rural communities.

### Data Sources and Processing

Information on vaccination status, demographic information, medical comorbidities, and frequency of medical visits were obtained from medical records. Spatial data were obtained from the census [26, 27]. We utilized the Area of Deprivation Index (ADI), which ranks neighborhoods based on socioeconomic factors such as income, education, employment, and housing quality from the Neighborhood Atlas [28, 29]. Patient satisfaction was extracted from standardized patient experience surveys. R software was used for statistical analysis (version 4.3.2) [30].

### Variable Definitions

**Figure 1.**
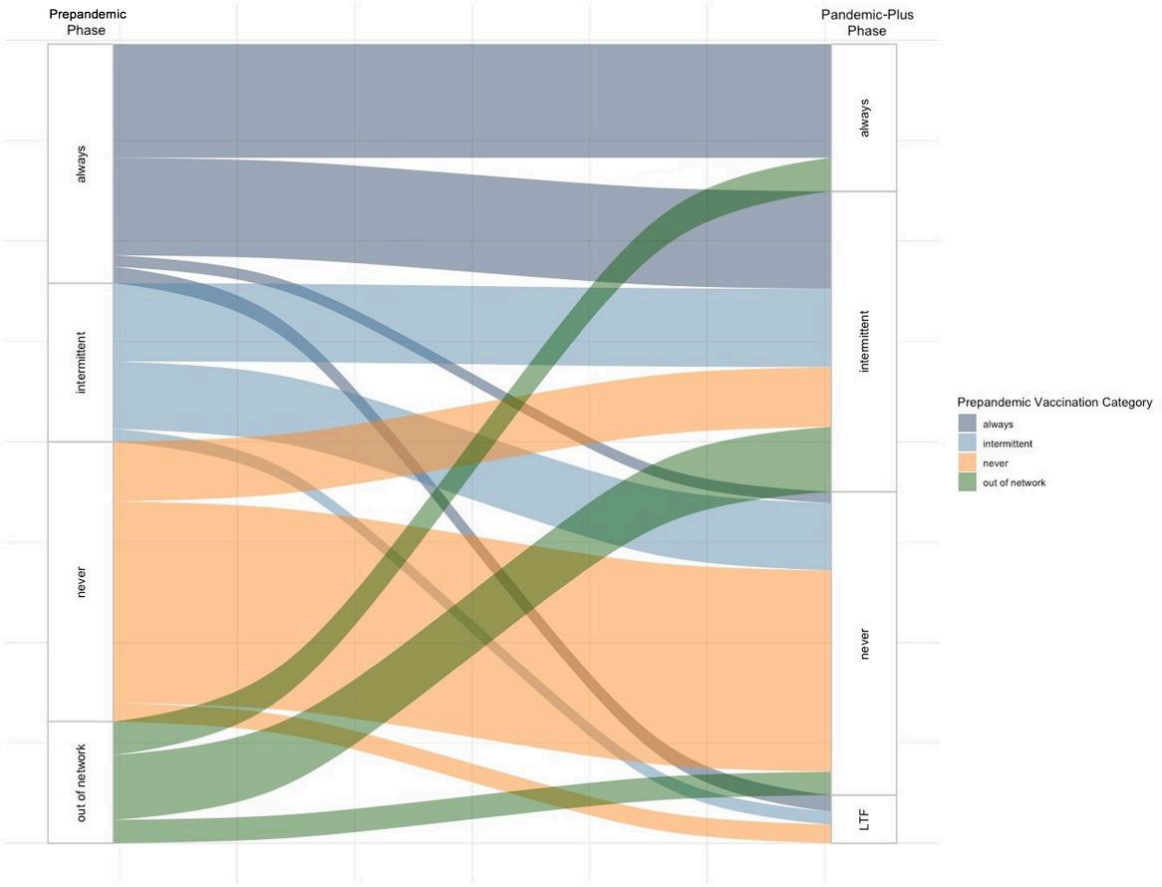
Flow diagram of the vaccination status of study participants (N = 173 825) from the Prepandemic phase (1 January 2017–28 February 2020) to the Pandemic-Plus phase (1 March 2020–31 December 2023). “Out of network” refers to patients who saw an out-of-network primary care provider during the Prepandemic phase but who established with a primary care provider during the Pandemic-Plus phase; “LTF” refers to patients who were lost to follow-up (eg, moved away or died) during the Pandemic-Plus phase. The figure represents the flow of participants such that the thickness of the lines corresponds to proportion of participants. There are instances where the number of participants who moved categories between pandemic phases were too small to be displayed in the figure given our large sample size (for example, among those who moved from the intermittent to the always category (n = 11).

Vaccination history was examined during 2 distinct 3-year phases: Prepandemic (1 January 2017–28 February 2020) and Pandemic-Plus (1 March 2020–31 December 2023). The division reflects the initial emergence of COVID-19 in the region [31]. Vaccination status for seasonal influenza was defined as “always vaccinated” (AV), “never vaccinated” (NV), or “intermittently vaccinated” (IV), referring to patients who are inconsistently vaccinated from year to year. An individual's vaccination status was defined both during the Prepandemic phase and, separately, during the Pandemic-Plus phase (Figure 1). Healthcare engagement was defined as having at least 1 primary care visit every 2 years, aligning with the timeframe after which a patient would be classified as a new patient for billing purposes. We calculated the Charlson Comorbidity Index (CCI) score [32, 33]. We applied the revised weights to reflect recent advancements in medical management since the 1980s [34, 35]. Due to relatively high levels of healthcare satisfaction, patients were classified as “satisfied” if they gave the maximum satisfaction rating for overall quality of care on standard patient experience surveys. Any rating below the maximum was categorized as “dissatisfied.”

### Spatiotemporal Assessment

We conducted a spatiotemporal assessment to examine the geographic distribution of the ADI and vaccination quartiles. Geographic data were visualized using mapping techniques described in our Supplementary Methods. Temporal trends were assessed by plotting the annual number of vaccination refusals, administrations, and overall patient satisfaction, stratified by vaccination category.

### Statistical Assessment, Univariate Logistic Regression, and Multivariable Logistic Regression

We compared demographic characteristics across the categorical vaccination categories using χ^2^ tests with continuity correction. Variables included individual characteristics (sex, age, ethnicity, preexisting medical conditions), ADI, patient satisfaction, and healthcare engagement. As this analysis was exploratory, adjustments for multiple comparisons were not applied.

Univariate logistic regression analyses were conducted to prioritize variables based on prehypothesized relationships. The following variables were included in the univariate analysis: ADI, highest level of education attainment, self-identified race, healthcare disengagement, patient satisfaction, survey completion, and CCI score.

For the multivariable model, we excluded colinear exposure variables based on independence testing (Supplementary Methods). The final model included healthcare disengagement, ADI, and CCI score.

### Patient Satisfaction Subanalysis

Only 43% of participants in our study completed standardized patient experience questionnaires. We found the distribution of the main predictors to be relatively similar between our subset and the entire study population (Supplementary Methods; Supplementary Material 1 and 2) and proceeded with multivariable model development.

The final model included healthcare disengagement, ADI, CCI score, and patient dissatisfaction. An interaction term between dissatisfaction and disengagement was incorporated to account for the hypothesized effect modification of this relationship.

### Interrupted Time Series Analysis

We summed the total monthly seasonal influenza vaccinations during our study period and fit an autoregressive integrative moving average (ARIMA) interrupted time series model [36]. Additional details on model development are available in the Supplementary Methods. We plotted the values predicted by our ARIMA model in the absence of the COVID-19 pandemic (the counterfactual) compared to the observed vaccination rate.

## RESULTS

### Descriptive Statistics

From 1 January 2017 to 31 December 2023, 173 825 primary care patients were included in our study (Table 1). Prior to the pandemic, 35.2% of individuals were AV (n = 51 773), 23.4% were IV (n = 34 310), and 41.3% were NV (n = 60 582) for seasonal influenza. Post–pandemic onset, 7.0% (n = 3556) of the Prepandemic AV and 48.7% (n = 16 710) of the Prepandemic IV never received another influenza vaccine (Table 1; Figure 1). A greater proportion of female participants and individuals with advanced educational degrees were AV during both phases. We observed a rise in the proportion of individuals with a high school level of education or less who were IV or NV during the Pandemic-Plus phase. The NV tended to have a higher ADI and lower healthcare engagement. We observed higher levels of dissatisfaction among the AV Prepandemic group and among the NV in the Pandemic-Plus group. Prepandemic, a greater proportion of individuals with severe CCI scores were AV; in the Pandemic-Plus phase, a greater proportion of these individuals were IV or NV (Table 1).

**Table 1.** Study Demographics From 2017 to 2023 (N = 173 825) Among Persons Who Were Always Vaccinated, Intermittently Vaccinated, or Never Vaccinated for Seasonal Influenza in the Prepandemic Phase (1 January 2017–28 February 2020) and the Pandemic-Plus Phase (1 March 2020–31 December 2023)

Characteristic	Prepandemic Phase				Pandemic-Plus Phase			
	Always	Intermittent	Never	*P* Value	Always	Intermittent	Never	*P* Value
	(n = 51 773)	(n = 34 310)	(n = 60 582)		(n = 31 952)	(n = 69 005)	(n = 72 868)	
Age, y, mean (SD)	60.9 (18.5)	54.0 (18.0)	50.7 (17.9)	<.001	59.1 (18.1)	55.7 (18.7)	51.5 (18.9)	<.001
Male sex	20 051 (38.7)	15 958 (46.5)	32 924 (54.3)	<.001	11 569 (36.2)	31 320 (45.4)	37 895 (52.0)	<.001
Self-identified race				<.001				<.001
Asian or Pacific Islander	1923 (3.7)	1434 (4.2)	2397 (4.0)		1550 (4.9)	2757 (4.0)	3253 (4.5)	
Black, African American, or African	935 (1.8)	1428 (4.2)	3425 (5.7)		558 (1.7)	2692 (3.9)	3885 (5.3)	
White	47 763 (92.3)	30 143 (87.9)	51 352 (84.8)		29 162 (91.3)	61 363 (88.9)	61 688 (84.7)	
Multiracial, other, or unknown	1152 (2.2)	1305 (3.8)	3408 (5.6)		682 (2.1)	2193 (3.2)	4042 (5.5)	
Highest level of education				<.001				<.001
High school or less	11 750 (26.1)	7430 (29.3)	13 005 (43.2)		6090 (20.7)	18 819 (34.0)	13 898 (37.7)	
Associate’s or bachelor's degree	23 235 (51.7)	13 235 (52.1)	14 002 (46.5)		15 165 (51.6)	27 792 (50.1)	17 470 (47.4)	
Master, doctorate, or professional degree	9977 (22.2)	4729 (18.6)	3124 (10.4)		8157 (27.7)	8812 (15.9)	5512 (14.9)	
Residing in an area with low deprivation^a^	25 704 (49.6)	17 383 (50.7)	21 137 (34.9)	<.001	17 311 (54.2)	29 587 (42.9)	27 125 (37.2)	<.001
Patients engaged in primary care services^b^	32 704 (63.2)	14 598 (42.5)	19 519 (32.2)	<.001	21 750 (68.1)	39 528 (57.30)	24 493 (33.6)	<.001
Influenza vaccination status, Prepandemic phase								<.001
Always	…	…	…		24 680 (100.0)	23 537 (43.4)	3556 (5.3)	
Intermittent	…	…	…		11 (0.0)	17 589 (32.3)	16 710 (24.7)	
Never	…	…	…		0 (0.0)	13 289 (24.4)	47 293 (70.0)	
Influenza vaccination status, Pandemic-Plus phase				<.001				
Always	24 680 (47.7)	11 (0.0)	0 (0.0)		…	…	…	
Intermittent	23 537 (45.5)	17 589 (51.3)	13 289 (21.9)		…	…	…	
Never	3556 (6.9)	16 710 (48.7)	47 293 (78.1)		…	…	…	
COVID-19 vaccination status				<.001				<.001
Primary series and boosters	42 950 (83.0)	21 649 (63.1)	19 879 (32.8)		30 285 (94.8)	48 303 (70.0)	25 685 (35.2)	
Primary series only	4618 (8.9)	4957 (14.4)	11 842 (19.5)		1294 (4.0)	10 466 (15.2)	13 258 (18.2)	
Incomplete primary series	925 (1.8)	1475 (4.3)	3097 (6.4)		241 (0.8)	5015 (7.3)	3011 (4.1)	
Unvaccinated	3280 (6.3)	6229 (18.2)	24 954 (41.2)		132 (0.4)	5221 (7.6)	30 914 (42.4)	
Dissatisfaction with overall healthcare^c^								
Prior to the pandemic	10 053 (19.4)	4404 (12.8)	3630 (6.0)	<.001	5974 (18.7)	8656 (12.5)	5355 (7.3)	<.001
After the pandemic	43 606 (84.2)	31 014 (90.4)	57 004 (94.1)	<.001	26 273 (82.2)	60 808 (88.1)	68 558 (94.1)	<.001
CCI score^d^				<.001				<.001
0 (mild)	28 262 (54.6)	22 091 (64.4)	44 635 (73.7)		18 596 (58.2)	42 297 (61.3)	51 948 (71.3)	
1–2 (moderate)	14 155 (27.3)	7948 (23.2)	10 872 (17.9)		8685 (27.2)	17 077 (24.7)	13 217 (18.1)	
>2 (severe)	9356 (18.1)	4271 (12.4)	5075 (8.4)		4671 (14.6)	9631 (14.0)	7703 (10.6)	

Data are presented as No. (%) unless otherwise indicated. *P* values were determined based on χ^2^ test with continuity correction.

Abbreviations: CCI, Charlson Comorbidity Index; COVID-19, coronavirus disease 2019; SD, standard deviation.

^a^Area of Deprivation Index (ADI) ranks neighborhoods by socioeconomic disadvantage such that a high ADI corresponds to high deprivation.

^b^Individuals were considered engaged in the healthcare system if they had 1 primary care appointment every 2 years after establishing care.

^c^Dissatisfied with overall care represents patients who were not maximally satisfied with their overall care after any interaction with the healthcare system.

^d^CCI is a weighted index that predicts the risk of death within 1 year based on several comorbid health conditions.

### Spatiotemporal Assessment

Patients from areas with higher quartiles of vaccinations tended to reside in areas closer to the hospital (Supplementary Material 3*A* and 3*B*), which tended to correspond to areas with lower ADI (Supplementary Material 3*B*).

**Figure 2.**
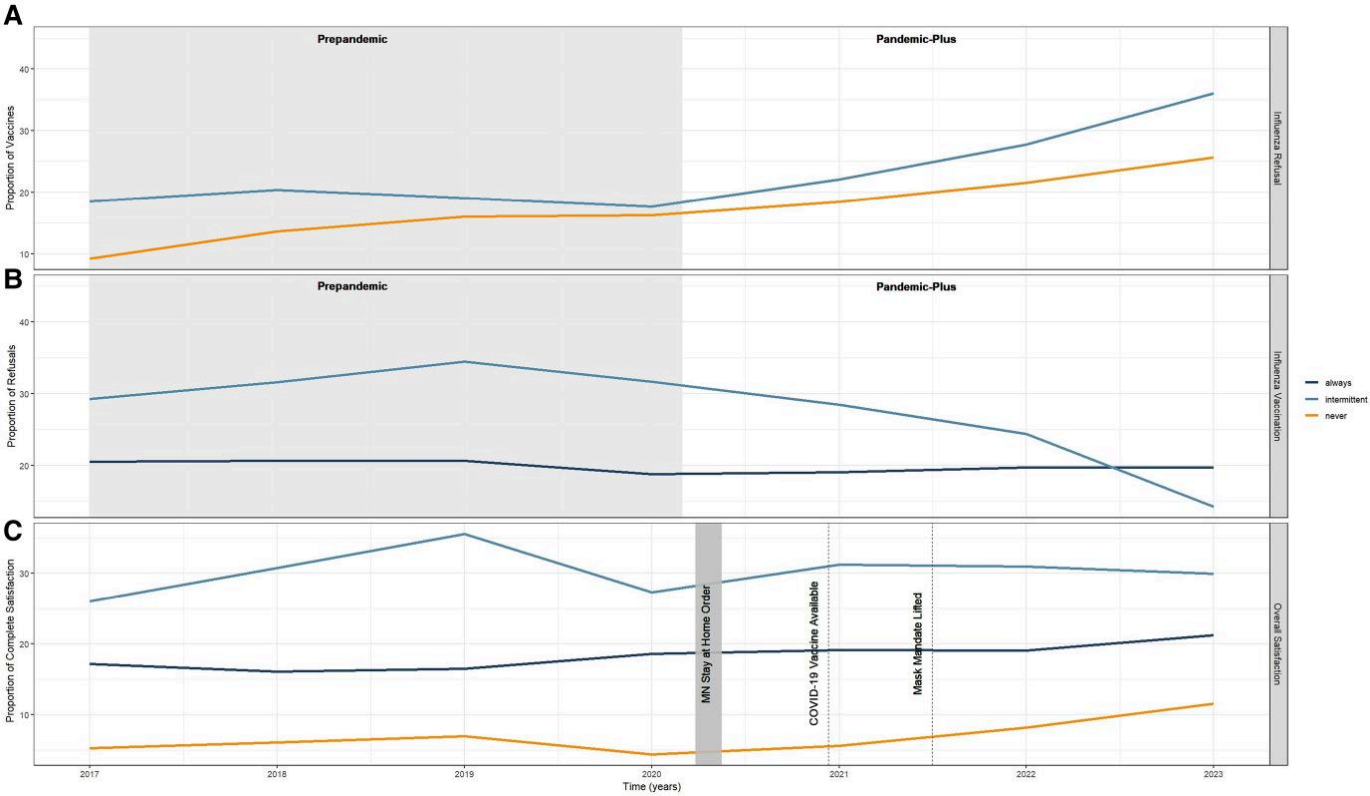
Yearly influenza vaccine refusal, influenza vaccine administrations, and overall patient satisfaction since establishing care with a primary care provider from 2017 to 2023. *A*, Yearly proportion of influenza vaccine refusals among individuals who are intermittently vaccinated and never vaccinated for seasonal influenza since establishing care with a primary care provider over time, where the denominator represents the total number of patients per year. *B*, Yearly proportion of vaccines administered over time since establishing care with a primary care provider among those who are always vaccinated and intermittently vaccinated for seasonal influenza, where the denominator represents the total number of primary care patients per year regardless of vaccination status. *C*, Yearly proportion of patients who indicated that they were completely satisfied with their overall care following any interaction with the healthcare system over time, where the denominator represents the total number of patients responding to satisfaction surveys per year. Abbreviations: COVID-19, coronavirus disease 2019; MN, Minnesota.

We observed a rise in the number of influenza vaccine refusals and a decline in the number of influenza vaccines administered following the onset of the COVID-19 pandemic (Figure 2). The proportion of vaccine refusals was greatest among the IV (Figure 2*A*) and corresponds to the decrease in vaccines administered being primarily driven by the IV (Figure 2*B*). We observed a relatively constant rate of vaccines administered among the AV across the 2 phases (Figure 2*B*). Lower satisfaction ratings were observed among the NV and highest among the IV (Figure 2*C*). Near the onset of the pandemic, we saw a decline in satisfaction among the IV and NV and a mild increase in satisfaction observed among the AV (Figure 2*C*).

### Univariate Logistic Regression

In the unadjusted logistic regression models during both phases, the NV had a higher odds ratio (OR) of residing in areas with greater ADI, having a high school level of education or less, self-identifying as Black or other racial minorities, and being disengaged compared to the AV (Supplementary Material 5). Those with advanced educational degrees, who were engaged in the healthcare system, completed satisfaction surveys, and had moderate or severe health comorbidities had a higher OR of being AV compared to the IV and NV throughout both phases (Supplementary Material 5). Additional details are provided in the Supplementary Results.

### Multivariable Logistic Regression

In the adjusted logistic regression model, there was a higher OR of healthcare disengagement among the IV or NV compared to the AV across phases, after adjusting for ADI and CCI score (Table 2, model 1). Between the 2 phases, this OR increased among the NV versus AV but decreased among the IV versus AV. Holding all else constant, individuals who were NV were more likely to have a higher ADI (Prepandemic: OR, 2.13 [95% confidence interval {CI}, 2.07–2.18]; Pandemic-Plus: OR, 2.27 [95% CI, 2.21–2.34]) compared to the AV. A similar relationship was observed when comparing the IV to AV in the Pandemic-Plus phase (OR, 1.64 [95% CI, 1.60–1.68]). Those with a moderate or severe CCI score were more likely to be AV (Table 2, model 1).

**Table 2.** Multinomial Logistic Regression (Adjusted Models) From the Cohort of Patients Participating in the Study (N = 173 825) (Model 1) and From a Subanalysis of Patients Who Responded to Standardized Patient Experience Questionnaires (n = 76 375) (Model 2)

Explanatory Variable	Prepandemic Phase^a^				Pandemic-Plus Phase^a^			
	Intermittent vs Always		Never vs Always		Intermittent vs Always		Never vs Always	
	OR (95% CI)	*P* Value	OR (95% CI)	*P* Value	OR (95% CI)	*P* Value	OR (95% CI)	*P* Value
Model 1								
Not engaged	2.20 (2.14–2.26)	<.001	3.33 (3.24–3.41)	<.001	1.63 (1.59–1.68)	<.001	4.23 (4.10–4.35)	<.001
ADI (ref = low)^b^	1.04 (1.01–1.07)	.007	2.13 (2.07–2.18)	<.001	1.64 (1.60–1.68)	<.001	2.27 (2.21–2.34)	<.001
Moderate CCI score (ref = mild)	0.83 (.80–.85)	<.001	0.57 (.56–.59)	<.001	0.91 (.88–.94)	<.001	0.65 (.63–.68)	<.001
Severe CCI score (ref = mild)	0.71 (.69–.74)	<.001	0.43 (.41–.44)	<.001	0.97 (.93–1.01)	.10	0.78 (.74–.81)	<.001
Model 2								
Dissatisfaction	0.93 (.89–.99)	.015	0.71 (.67–.75)	<.001	1.11 (1.06–1.17)	<.001	1.25 (1.18–1.33)	<.001
Not engaged	1.94 (1.85–2.03)	<.001	2.59 (2.47–2.71)	<.001	1.67 (1.55–1.81)	<.001	3.01 (2.76–3.29)	<.001
Dissatisfaction × engagement	1.03 (.95–1.13)	.5	1.18 (1.08–1.30)	.02	0.84 (.77–.92)	<.001	1.02 (.93–1.13)	.6
ADI (ref = low)^b^	0.99 (.95–1.03)	.7	1.92 (1.84–1.99)	<.001	1.53 (1.47–1.58)	<.001	1.81 (1.74–1.88)	<.001
Moderate CCI score (ref = mild)	0.86 (.82–.90)	<.001	0.80 (.76–.84)	<.001	0.99 (.95–1.03)	.6	0.88 (.84–.92)	<.001
Severe CCI score (ref = mild)	0.78 (.74–.82)	<.001	0.73 (.69–.77)	<.001	1.18 (1.13–1.24)	<.001	1.21 (1.14–1.27)	<.001

Abbreviations: ADI, Area of Deprivation Index; CCI, Charlson Comorbidity Index; CI, confidence interval; OR, odds ratio.

^a^“Prepandemic phase” refers to influenza immunization data and patient satisfaction data prior to March 2020. “Pandemic-Plus phase” refers to immunization data and patient satisfaction data from March 2020 to January 2024.

^b^Low ADI ranking refers to areas with a low level of deprivation, whereas high ADI ranking refers to areas with a high level of deprivation. Individuals were considered to have low ADI if they came from an area with a state ADI ranking from 1 to 5 and were considered to have high ADI if they came from an area with a state ADI ranking from 6 to 10.

### Patient Satisfaction Subanalysis

Within the subset of patients who completed standardized patient experience questionnaires (n = 76 375; Supplementary Results and Supplementary Material 1 and 2), in the Prepandemic phase we observed a lower OR of dissatisfaction among the IV or NV versus the AV after adjusting for healthcare engagement, ADI, and CCI score (Table 2, model 2). Following the COVID-19 pandemic, we observed a higher OR of dissatisfaction among the IV or NV versus AV, holding all else constant. There was a significant interaction between dissatisfaction and disengagement among the NV versus AV in the Prepandemic phase (*P* < .001) and among the IV versus AV in the Pandemic-Plus phase (*P* < .001).

Holding all else constant, the NV were more likely to have a higher ADI (Prepandemic: OR, 1.92 [95% CI, 1.84–1.99]; Pandemic-Plus: OR, 1.81 [95% CI, 1.74–1.88]) compared to the AV. A similar relationship was observed among the IV versus AV in the Pandemic-Plus phase (OR, 1.53 [95% CI, 1.47–1.58]) but was not significant Prepandemic. In the Prepandemic phase, those with severe comorbidities were less likely to be IV (OR, 0.78 [95% CI, .74–.82]) or NV (OR, 0.73 [95% CI, .69–.77]) compared to the AV, holding all else constant. However, in the Pandemic-Plus phase, these individuals had a higher odds of being IV (OR, 1.18 [95% CI, 1.13–1.24]) or NV (OR, 1.21 [95% CI, 1.14–1.27]) versus AV after adjusting for the other covariates.

### Interrupted Time Series Analysis

In our ARIMA model assessing vaccination rates over time, the null hypothesis that the time series was a purely random process was rejected (χ^2^ = 15.23, *P* = .91). Based on prior trends, our model predicted higher rates of vaccination in the instance that the COVID-19 pandemic did not occur (Supplementary Material 6). We observe a declining trend of patient dissatisfaction across all categories and study phases (Supplementary Material 7).

## DISCUSSION

We conducted a retrospective cohort analysis of adult primary care patients to assess the impact of the COVID-19 pandemic on influenza vaccination uptake. Our primary finding was a decline in vaccination rates following the pandemic onset, most pronounced among IV individuals (Figures 1 and 2). In our multivariable model, we observed a higher odds of healthcare disengagement among the IV and NV compared to the AV throughout all phases (Table 2, model 1). In a subgroup who participated in postvisit surveys, there was a higher OR of healthcare dissatisfaction among the IV and NV compared to the AV in the Pandemic-Plus phase (Table 2, model 2). After adjusting for patient satisfaction, healthcare engagement, and ADI, we estimate that those with severe health comorbidities were less likely to be consistently vaccinated following the COVID-19 pandemic. Interestingly, these individuals were more likely to be AV Prepandemic (Table 2, model 2).

The decline in vaccination rates following the COVID-19 pandemic has raised concerns, particularly for individuals at elevated risk of severe influenza-related complications. Early in the pandemic, refusals may have been driven by missed opportunities for vaccination [37], for example, due to social distancing or lack of access. Others have hypothesized that increased media focus on COVID-19 may have overshadowed the importance of influenza and underestimation of influenza-related complications [38]. This belief was likely augmented by the widespread behavioral measures that decreased viral circulation, further contributing to lower perceived infection risk [38, 39]. However, declining rates of influenza vaccination have persisted for multiple years following emergence of SARS-CoV-2 (Figure 2; Supplementary Material 6), suggesting that the pandemic has fundamentally shifted vaccination behaviors.

In our study, the observed shift in vaccination practice may be attributed to the growing odds of healthcare disengagement between pandemic phases, particularly among the NV (Table 2). Interestingly, while the odds of disengagement remained significantly elevated among the IV following the pandemic, we observed a decline in OR when compared to the Prepandemic phase. This may be driven by the 2-fold increase in the number of IV participants in the Pandemic-Plus phase (Table 1). In our subanalysis, the IV and NV had lower odds of healthcare dissatisfaction Prepandemic and a higher odds following the pandemic compared to the AV (Table 2, model 2). These findings may be related to rising mistrust in the healthcare system and spread of health-related misinformation [40], resulting in decreased routine health appointments and, ultimately, vaccine administration. A meta-analysis found that trust in science and local health departments was predictive of decreased vaccine hesitancy [21]. This was echoed in our study as healthcare satisfaction increased among the AV (Supplementary Material 5; Figure 2) following the pandemic. Although our study did not directly measure trust in the healthcare system, the substantial and increasing levels of healthcare disengagement and dissatisfaction warrant further investigation. These findings raise questions about the efficacy of healthcare-led interventions to improve vaccination efforts and suggest the need to explore alternative strategies targeting disengaged populations and to maintain positive patient engagement.

The decision to receive routine vaccinations is likely multifactorial. Influenza vaccination has previously been associated with location and economic status [13], but such sociodemographic variables are inconsistent predictors [22] due to regional differences. In our analysis, individuals who self-identify as Black were less likely to be consistently vaccinated across study phases (Supplementary Material 5). Historical inequities in healthcare access and disparities in health outcomes have disproportionately affected people of color in the United States and resulted in limited access to preventive healthcare services, including vaccinations [41–43]. Similar findings have been reported among other racial minorities [44, 45], which was also observed in our study (Supplementary Material 5). Moreover, our participants residing in areas with high ADI and with lower levels of educational attainment were less likely to be consistently vaccinated (Supplementary Material 5; Table 1). This echoes prior studies that have found associations between reduced vaccine hesitancy with higher economic status [21] and higher educational attainment [19]. Medical providers and public health officials should be aware of differential levels of vaccination hesitancy based on sociodemographic factors to tailor future vaccination initiatives.

Additionally, individuals with elevated health comorbidity scores were more likely to be AV (Table 2, model 1) across study phases. However, in the subanalysis, during the Pandemic-Plus phase, individuals with severe comorbidities had higher odds of being IV or NV after adjusting for dissatisfaction, disengagement, and ADI (Table 2, model 2). This difference could be attributed to the higher proportional representation of health comorbidities in the subset (Supplementary Material 1); however, it is unclear if the observed magnitude of difference can be fully attributed to this approximately 10% difference in representation. It is equally possible that healthcare dissatisfaction is a key driver in the decision to become vaccinated among patients with severe comorbidities. Following the pandemic, other researchers have raised similar concerns about rising vaccine hesitancy among those with severe comorbidities—including solid organ transplant recipients [46], people with multiple sclerosis [47], elderly persons [48], and people with human immunodeficiency virus [49]. Others identified that individuals with high levels of perceived risk were more likely to engage in information-seeking approaches to inform vaccination behaviors following the pandemic [48]. In our study, individuals who were less likely to be consistently vaccinated were also the least engaged in the healthcare system (Table 2, model 1; Supplementary Material 1). These findings raise concerns about where individuals—particularly those who may benefit most from vaccination—are obtaining vaccine-related information, and the accuracy of these sources.

This study has several limitations. We included all primary care patients in the system catchment area, which includes hospital employees. It is unclear how many participants received employer-mandated vaccines immediately following the COVID-19 pandemic and how this may shape future behaviors. A meta-analysis found that, while overall healthcare workers were receptive of mandatory vaccines, there were variations by profession [50]. Our study did not collect information about employer-mandated vaccinations. Additionally, the Mayo Clinic Health System is a quaternary medical center and the population may skew toward patients with greater health comorbidities, which may influence healthcare motivation and perceived infection risk. Moreover, our study only includes patients with access to primary care and does not reflect the vaccination behaviors of those with limited healthcare access, such as migrant populations and ethnic minority groups. This likely contributes the overrepresentation of patients with self-identified White ethnicities. Additionally, this study only includes data from a single state and may not be generalizable to other locations with different demographic characteristics and political climates. Overall, our study had a very high level of patient satisfaction, which made it difficult to detect variability. Thus, we only considered patients reporting perfect satisfaction scores as “satisfied.” Given this strict definition, our study is not powered to detect the nuances of satisfaction that may impact willingness to receive vaccines and may not be generalizable to all medical settings. While many patients were encouraged to complete patient experience surveys, participation was voluntary. Thus, results may skew toward extreme experiences or those motivated to participate. Finally, in our ARIMA models, the predicted counterfactual calculation was based on the trends determined in the prior years and it is impossible to predict exactly what would have happened in the absence of the COVID-19 pandemic. Information on COVID-19 vaccinations is included in Supplementary Material 8 and 9.

## CONCLUSIONS

The COVID-19 pandemic changed vaccination behaviors and healthcare satisfaction, particularly among those with severe comorbidities. Decline in influenza vaccination is associated with residing in areas with high ADI, self-identifying as Black, low education attainment, and healthcare disengagement. Healthcare providers and public health officials should be aware of the nuanced factors associated with vaccine refusal to create targeted vaccine interventions.

## Supplementary Material

ofaf351_Supplementary_Data

## Supplementary Data

Supplementary materials are available at *Open Forum Infectious Diseases* online. Consisting of data provided by the authors to benefit the reader, the posted materials are not copyedited and are the sole responsibility of the authors, so questions or comments should be addressed to the corresponding author.

## Notes

***Author contributions*.** D. W. C. and J. C. H. were responsible for conceptualization and provision of resources. D. W. C., J. C. H., and O. M. M. were responsible for data curation and methodology. O. M. M. was responsible for the formal analysis and writing–original draft preparation. D. W. C., J. C. H., and J. W. M. assisted with supervision of the project. J. W. M., J. Y., L. L. W., L. H., T. W., J. C. H., and D. W. C. were responsible for writing–review and editing.

***Data availability*.** The data for this study are available under restricted access due to patient confidentiality. The raw data are protected and not available due to data privacy laws. Access to processed de-identified data may be available upon reasonable request to the corresponding author. Data are located in a controlled-access electronic storage system managed by the Mayo Clinic.

***Patient consent*.** All participants agreed to have their de-identified medical records used for research purposes. The study was approved by the Mayo Clinic Institutional Review Board.
