# Supplementary material for: The Influence of the Coronavirus Disease 2019 Pandemic on Influenza Vaccination Refusal and Patient Satisfaction
Source: Open Forum Infect Dis. 2025 Jun 18;12(7):ofaf351. doi: 10.1093/ofid/ofaf351 (PMC12236155; doi:10.1093/ofid/ofaf351)
Supplement: ofaf351_Supplementary_Data [file ofaf351_supplementary_data.docx]

**Supplemental Materials**

**Supplemental Methods:**

Spatial assessment: Maps were created using the ‘tigris’ R package,^1^ which allows direct shapefile download from the United States Census Bureau^2^. We created a map of Minnesota that outlines the Mayo Clinic Health System (MCHS) catchment level at the zip code level. We obtained the coordinates of the main Mayo Clinic Hospital, located in Rochester, MN, through geocoding the address. Next, the area of deprivation index (ADI) was mapped at the census block level, as recommended by Kind et al^3,4^. Finally, vaccination status was mapped at the 5-digit zip code level to preserve anonymity of our participants.

Assessment of collinearity: We examined potential colinear variables through chi-squared independence testing. Self-identified race and highest level of educational attainment were excluded from the final model due to significant collinearity with ADI (p<0.0001). Similarly, completion of surveys was excluded because of its high collinearity with health system engagement (p<0.0001).

Interrupted timeseries analysis: To create the Autoregressive Integrative Moving Average (ARIMA) model, we followed the methods outlined by Schaffer et al^5^ . First, seasonal influenza vaccination was modeled as a linear combination of vaccinations in the prior month. To assess the appropriateness of the model, we examined residual plots and formally tested for autocorrelation using the Ljung-Box test for white noise. The null hypothesis was that seasonal influenza vaccination was a random process without significant autocorrelation (p-value 0.91). Thus, we did not reject the null hypothesis, indicating our model has a good fit. We then plotted the values predicted by our ARIMA model in the absence of the COVID-19 pandemic (the counterfactual) compared to the observed vaccination rate (Figure 5).

Patient satisfaction sub-analysis distribution:

Forty three percent of participants in our study completed standardized patient experience questionnaires. To account for this limitation, we analyzed patient satisfaction exclusively within this subset of participants. Overall, we found the distribution of our main predictors between the entire study and the subset of participants who completed experience questionnaires to be relatively similar (Supplement S2-S3). However, the subset of patients who participated in patient experience questionnaires, did tend to have a greater proportion of individuals with moderate or severe health comorbidities when compared to the entire population (Figure S2, Table 1), but had a similar distribution of participants based on ADI (Supplement S3, Table 1).

**Supplemental Results:**

Univariate logistic regression:

In the unadjusted logistic regression models during the Pre-Pandemic and Pandemic-Plus phases, the NV had a higher odds ratio (OR) of residing in areas with greater deprivation [1.84 (1.80, 1.88); 1.99 (1.94, 2.05)], having a high school level of education or less [3.53 (3.37, 3.71); 3.38 (3.23, 3.53)], self-identify as Black [3.41 (3.17, 3.67); 3.29 (3.01,3.60)] or other racial minority [2.75 (2.57, 2.94); 2.80 (2.58,3.04)], and being disengaged with the healthcare system [3.61 (3.52, 3.70); 2.80 (2.58, 3.04)] compared to the AV (Supplement Table S1). In contrast, the NV were less likely to complete satisfaction surveys [0.16 (0.16, 0.17); 0.17 (0.16, 0.17)] versus the AV during both phases respectively (Table S1). Participants with moderate or severe health comorbidities had a lower likelihood of being IV or NV compared to the AV [Moderate vs Mild Pre-Pandemic 0.72 (0.70, 0.74), 0.49 (0.47, 0.50); Pandemic-Plus 0.86 (0.84, 0.89), 0.54 (0.53, 0.56); Severe vs Mild Pre-Pandemic 0.58 (0.56, 0.61), 0.34 (0.33, 0.36); Pandemic-Plus 0.91 (0.87, 0.94), 0.59 (0.57, 0.61), respectively]. Male gendered participants had a higher odds of being IV [Pre-Pandemic 1.38 (1.34, 1.41), Pandemic-Plus 1.46 (1.42, 1.50)] or NV [Pre-Pandemic 1.88 (1.84, 1.93), Pandemic-Plus 1.91 (1.86, 1.96)] versus the AV.

Compared to the AV, the IV had a lower OR of residing in an area with high deprivation Pre-Pandemic [0.96 (0.93, 0.99)] and a higher OR of residing in an area with high deprivation in the Pandemic-Plus phase [1.58 (1.53, 1.62)].

Those with advanced educational degrees, who were engaged in the healthcare system, completed satisfaction surveys, and had moderate or severe health comorbidities had a higher OR of being AV compared to the IV and NV throughout both phases (Table S1).

**Supplemental Tables and Figures**

| **Supplement S1:** Severity of health comorbidity distribution among participants who completed patient experience questionnaires compared to those who did not by vaccination category. |
| --- |
| 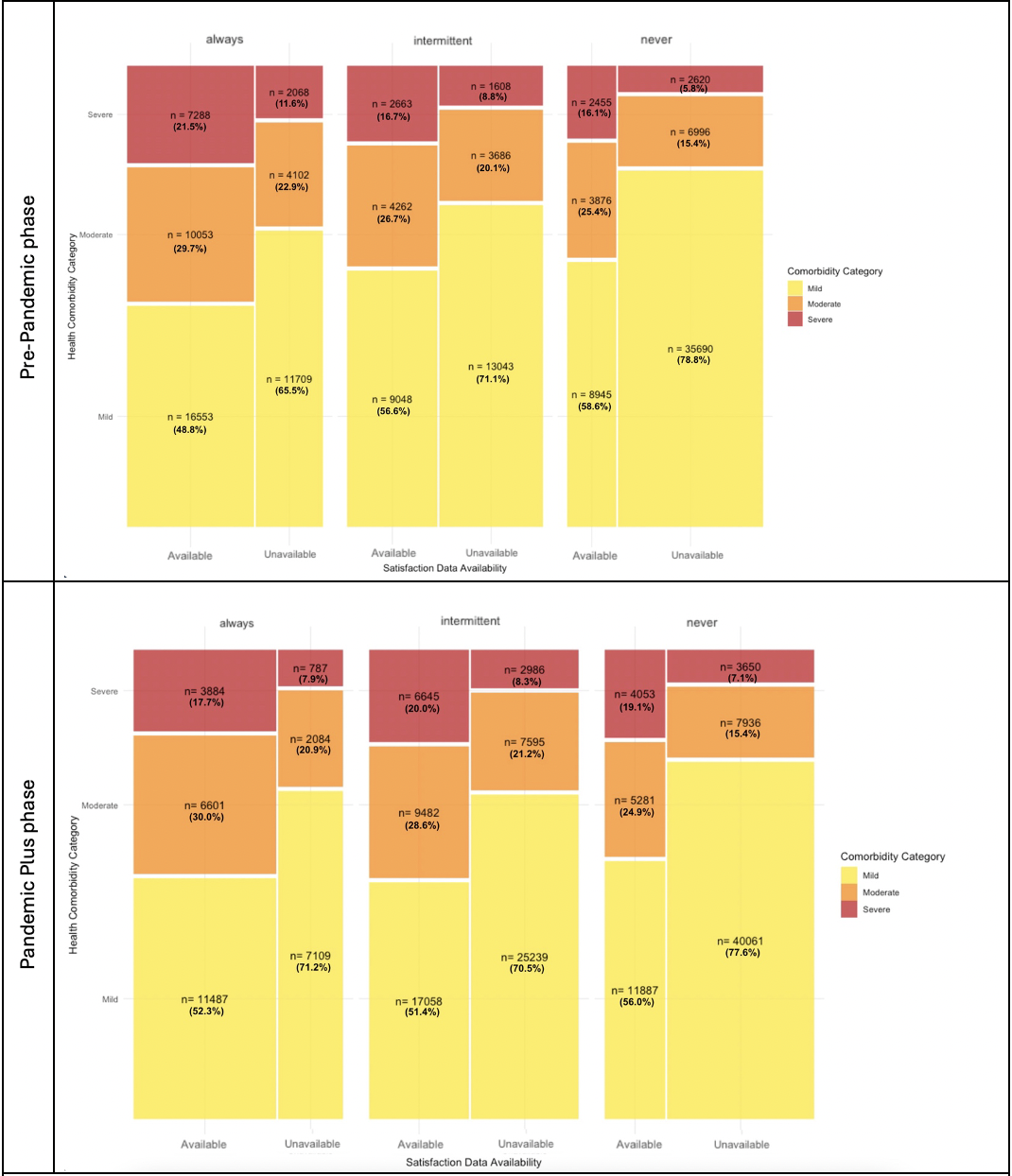 |
| Comorbidity category was determined based on the Charlson Comorbidity Index. Percentages depicted were calculated based on column values. |

| **Supplement S2:** Distribution of Area of deprivation among participants who completed patient experience questionnaires compared to those who did not by vaccination category. |
| --- |
| 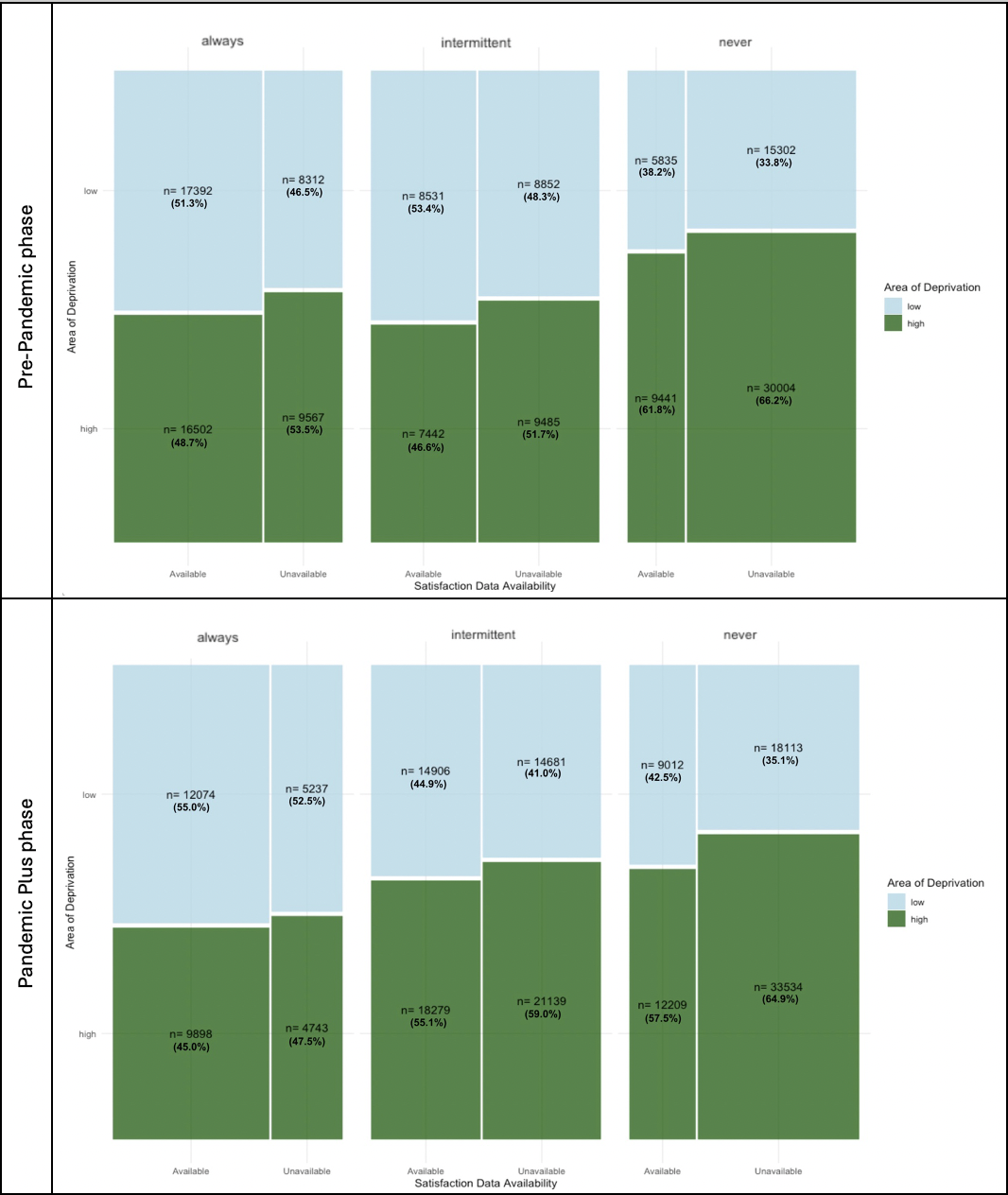 |
| Deprivation categories were determined based on the Area of Deprivation Index. Percentages depicted were calculated based on column values. |

| **Supplement S3:** Population adjusted maps of the quartiles of (A) individuals who are always vaccinated; (B) individuals who are intermittently vaccinated; and (C) individuals who are never vaccinated for seasonal influenza after establishing care with a primary care physician by 5-digit zip code. |
| --- |
| 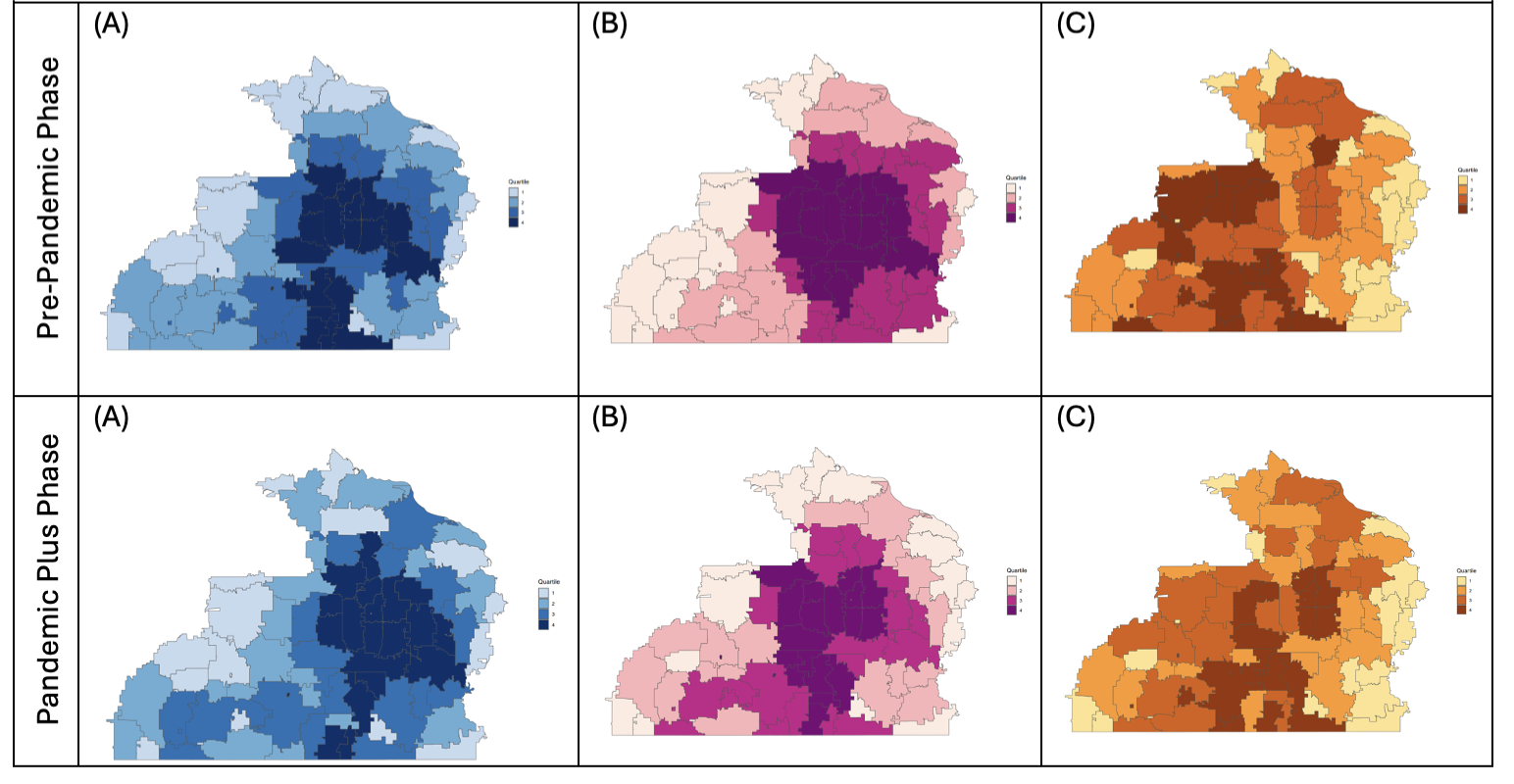 |
| Pre-Pandemic phase corresponds to dates between 1/1/2017 and 2/28/2020. The Pandemic-Plus phase corresponds to dates between 3/1/2020 and 12/31/2023.  The darker colors represent higher quartiles of vaccinations (A, B) and higher quartiles of refusals (C). |

| **Supplement S4:** Demographic maps displaying Minnesota with the health system catchment area (A) and the Area Deprivation Index (ADI, B) | |
| --- | --- |
| (A)   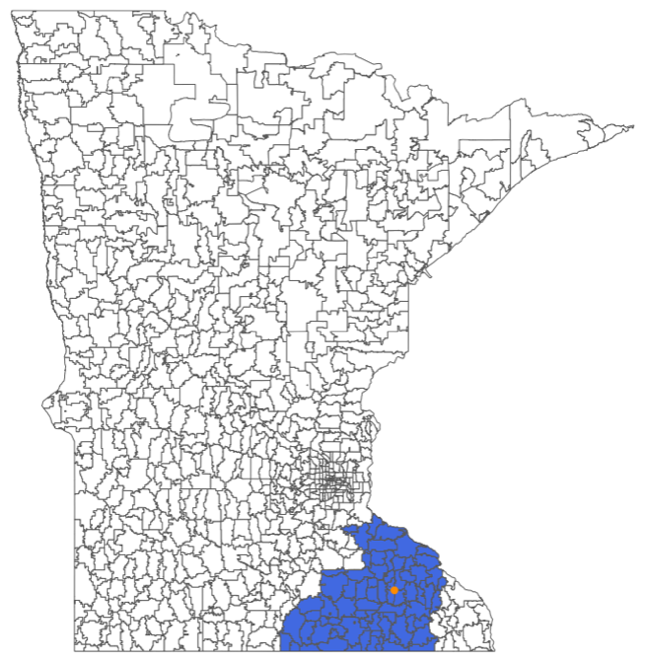 | (B)   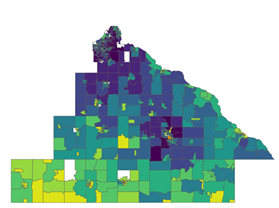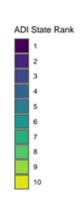 |
| (A) Map of the state of Minnesota with a shaded region corresponding the hospital catchment area. Orange coordinates represent the large quaternary care hospital found in the region.  (B) ADI state rank refers to percentile ranking by census block such that a score of 1 (darkest color) corresponds to areas with the lowest level of disadvantage and a score of 10 (yellow color) corresponds to areas with the highest level of disadvantage.  Orange coordinates represent the large quaternary care hospital found in the region. | |

| **Supplement S5:** Univariate logistic regression (unadjusted), n= 173,825. | | | | |
| --- | --- | --- | --- | --- |
|  | Pre-Pandemic Phase ^t^ | | Pandemic + Phase ^t^ | |
| Explanatory Variable | OR (95% CI) Intermittent vs. Always Vaccinated for Influenza | OR (95% CI)  Never vs. Always Vaccinated for Influenza | OR (95% CI) Intermittent vs. Always Vaccinated for Influenza | OR (95% CI)  Never vs. Always Vaccinated for Influenza |
| *Gender* | | | | |
| Female | ref | ref | ref | ref |
| Male | 1.38 (1.34, 1.41) | 1.88 (1.84, 1.93) | 1.46 (1.42, 1.50) | 1.91 (1.86, 1.96) |
| *Area of deprivation index (ADI)^^^* | | | | |
| Low | ref | ref | ref | ref |
| High | 0.96 (0.93, 0.99) | 1.84 (1.80, 1.88) | 1.58 (1.53, 1.62) | 1.99 (1.94, 2.05) |
| *Highest level of education* | | | | |
| Advanced degree | Ref | Ref | Ref | Ref |
| Associate or Bachelor’s degree | 1.20 (1.15, 1.25) | 1.92 (1.84, 2.01) | 1.70 (1.64, 1.76) | 1.70 (1.64, 1.78) |
| High school or less | 1.33 (1.28, 1.40) | 3.53 (3.37, 3.71) | 2.86 (2.74, 2.98) | 3.38 (3.23, 3.53) |
| *Self-Identified Race* | | | | |
| White | *Ref* | *Ref* | *Ref* | *Ref* |
| Black | 2.42 (2.23, 2.63) | 3.41 (3.17, 3.67) | 2.29 (2.09, 2.51) | 3.29 (3.01, 3.60) |
| Asian | 1.18 (1.10, 1.27) | 1.16 (1.09, 1.23) | 0.85 (0.79, 0.90) | 0.99 (0.93, 1.06) |
| Other Minority | 1.79 (1.66, 1.94) | 2.75 (2.57, 2.94) | 1.53 (1.40, 1.67) | 2.80 (2.58, 3.04) |
| *Charlson Comorbidity Index (CCI)* | | | | |
| Mild | ref | ref | ref | ref |
| Moderate | 0.72 (0.70, 0.74) | 0.49 (0.47, 0.50) | 0.86 (0.84, 0.89) | 0.54 (0.53,0.56) |
| Severe | 0.58 (0.56, 0.61) | 0.34 (0.33, 0.36) | 0.91 (0.87, 0.94) | 0.59 (0.57, 0.61) |
| *Engagement with the health care system^*^* | | | | |
| Engaged | ref | ref | ref | ref |
| Not engaged | 2.32 (2.25, 2.38) | 3.61 (3.52, 3.70) | 1.59 (1.55, 1.64) | 4.21 (4.09, 4.33) |
| *Satisfaction data* | | | | |
| Completes Surveys | 0.49 (0.48, 0.50) | 0.16 (0.16, 0.17) | 0.42 (0.41, 0.44) | 0.17 (0.16, 0.17) |
| *Satisfaction with overall care (n= 76,375) ^#^* | | | | |
| Not satisfied | 0.61 (0.59, 0.64) | 0.26 (0.25, 0.28) | 1.60 (1.55, 1.66) | 3.44 (3.30, 3.59) |
| t: The “Pre-pandemic phase” refers to influenza immunization data and patient satisfaction data prior to March 2020. The “Pandemic + phase” refers to immunization data and patient satisfaction data from March 2020 to January 2024.  ^: Low ADI ranking refers to areas with a low level of deprivation, whereas high ADI ranking refers to areas with a high level of deprivation. Individuals were considered low if they came from an area with a state ADI ranking from 1 to 5 and were considered high if they came from an area with a state ADI ranking from 6 to 10.  *: Engagement within the healthcare system refers to individuals who average one primary care appointment every two years since establishing care with a primary care physician  #: Not satisfied includes patients who reported any negative satisfaction score for the overall care received, within each time phase, during interactions with outpatient provider, inpatient hospitalization, emergency department, or surgery. Patients who were satisfied with the overall care received always reported the highest level of satisfaction for each interaction. | | | | |

| **Supplement S6:** ARIMA model of vaccination rate per 10,000 over time at the monthly resolution |
| --- |
| 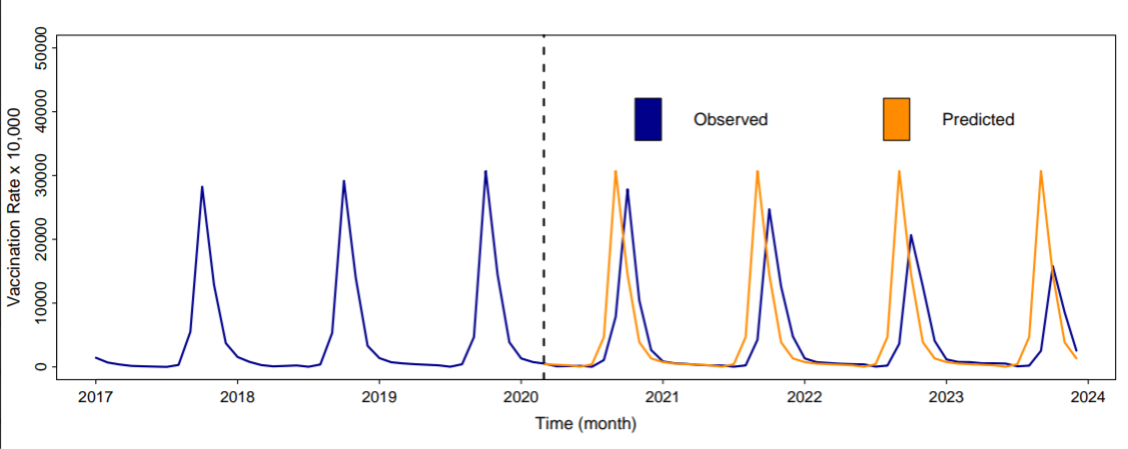 |
| The blue line corresponds to observed trend in vaccination rates over time. Orange lines correspond to predicted trends that would have occurred in the absence of the COVID-19 Pandemic (counterfactual). The dashed line corresponds to the onset of the COVID-19 Pandemic. |

| **Supplement S7:** Patient dissatisfaction x 10,000 over time at a monthly resolution |
| --- |
| 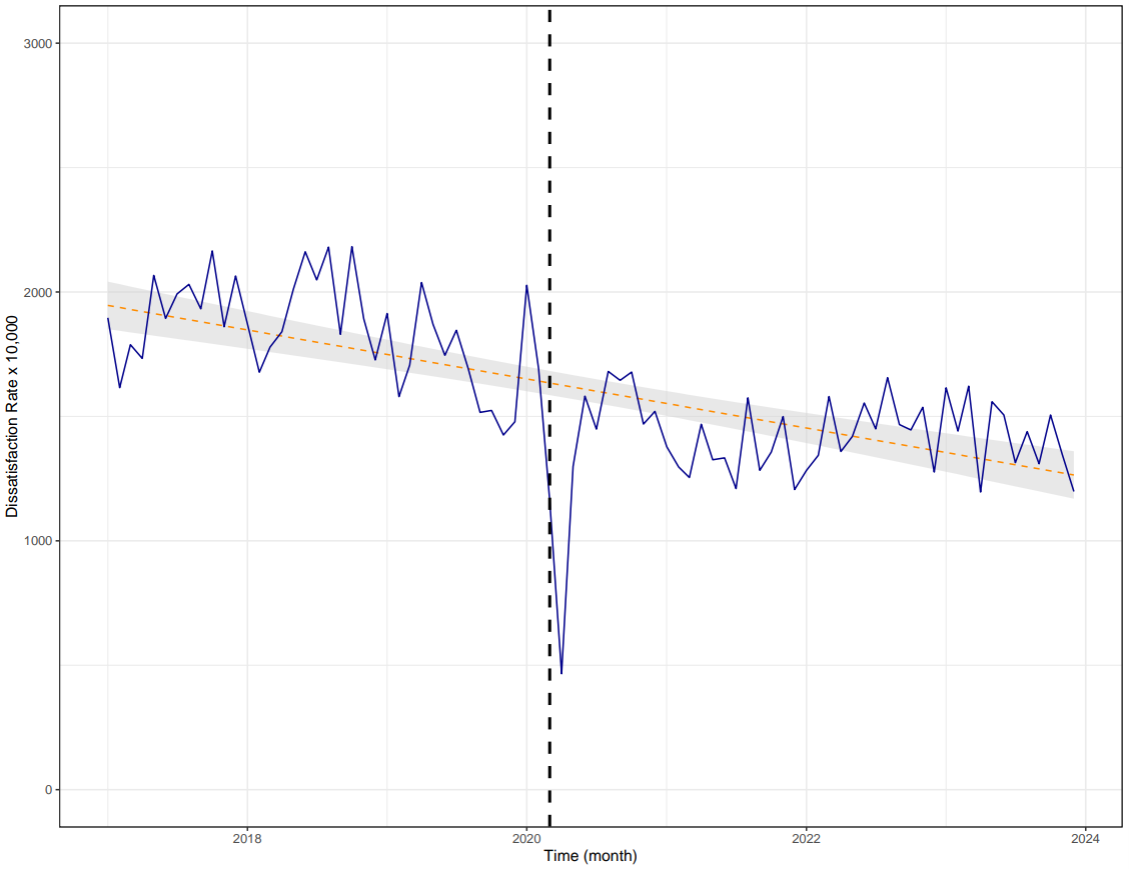 |
| The blue line corresponds to observed trend in dissatisfaction rates over time. Orange line corresponds to the estimated linear trend with a 95% confidence interval in grey. The black dashed line corresponds to the onset of the COVID-19 Pandemic. |


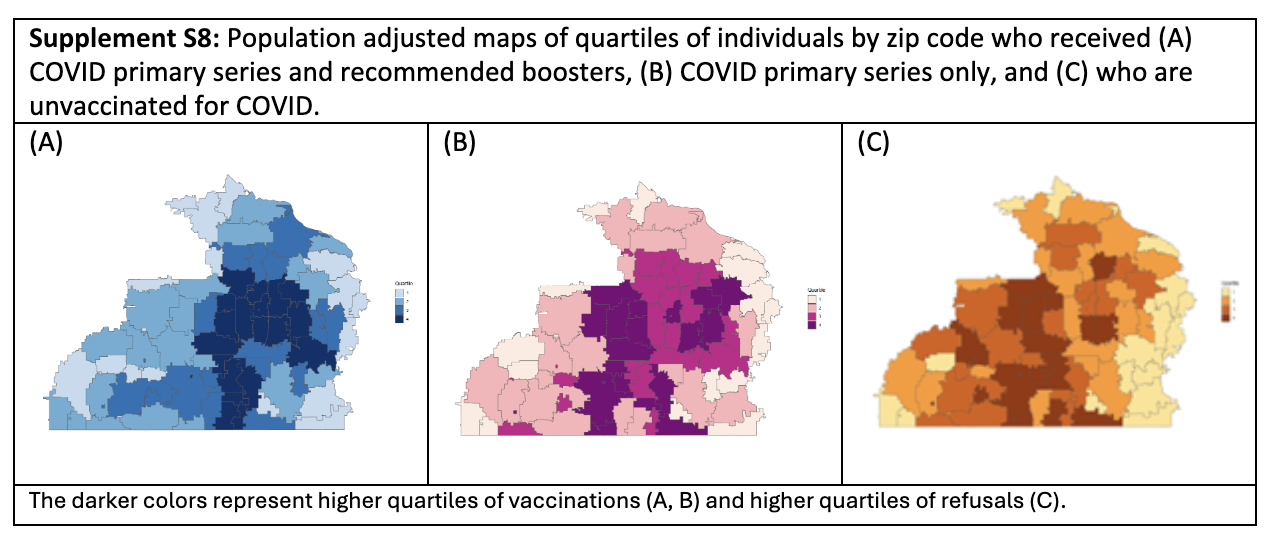


| **Supplement S9:** Number of patients who completed the primary COVID-19 vaccination series, who completed the primary COVID-19 vaccination series with two boosters, and who refused vaccination since establishing care with a primary care physician. |
| --- |
| 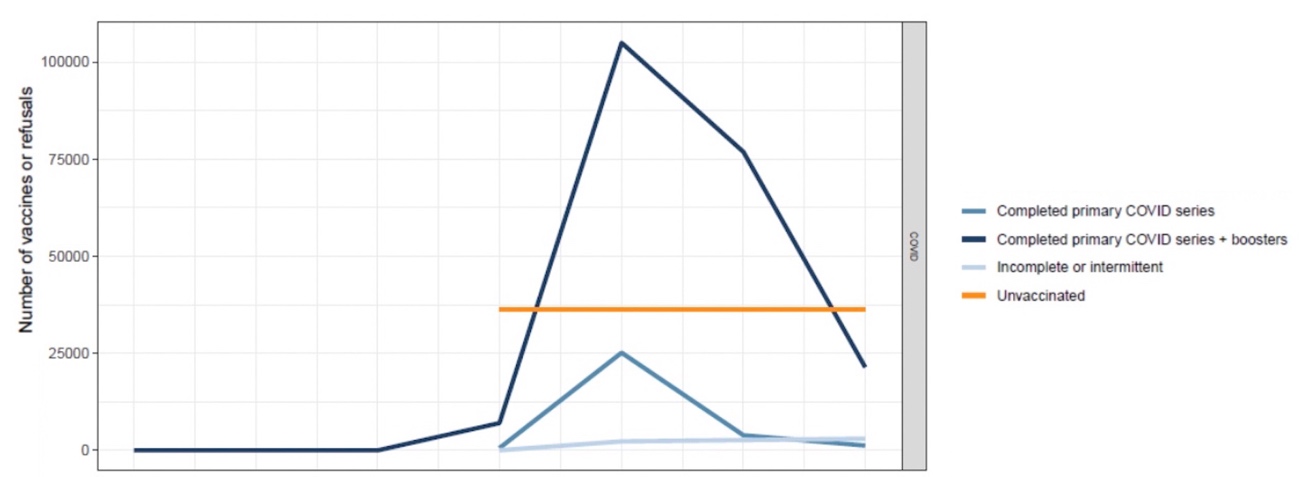 |

**References:**

1. Walker K. tigris: Load Census TIGER/Line Shapefiles. Published online 2022.

2. United States Census Bureau. Explore Census Data. https://data.census.gov/.

3. Kind AJH, Buckingham WR. Making Neighborhood-Disadvantage Metrics Accessible — The Neighborhood Atlas. *New England Journal of Medicine*. 2018;378(26):2456-2458. doi:10.1056/NEJMp1802313

4. University of Wisconsin School of Medicine Public Health. Area Deprivation Index v2.0. . https://www.neighborhoodatlas.medicine.wisc.edu/ May 23, 2019.

5. Schaffer AL, Dobbins TA, Pearson SA. Interrupted time series analysis using autoregressive integrated moving average (ARIMA) models: a guide for evaluating large-scale health interventions. *BMC Med Res Methodol*. 2021;21(1):58. doi:10.1186/s12874-021-01235-8
